# Social density, but not sex ratio, drives ecdysteroid hormone provisioning to eggs by female house crickets (*Acheta domesticus*)

**DOI:** 10.1002/ece3.4502

**Published:** 2018-10-02

**Authors:** Katherine C. Crocker, Mark D. Hunter

**Affiliations:** ^1^ Department of Environmental Health Sciences Mailman School of Public Health at Columbia University New York New York; ^2^ Department of Ecology and Evolutionary Biology University of Michigan Ann Arbor Michigan

**Keywords:** density dependence, hormone provisioning, maternal effects, non‐Mendelian parental effects

## Abstract

Social environment profoundly influences the fitness of animals, affecting their probability of survival to adulthood, longevity, and reproductive output. The social conditions experienced by parents at the time of reproduction can predict the social environments that offspring will face. Despite clear challenges in predicting future environmental conditions, adaptive maternal effects provide a mechanism of passing environmental information from parent to offspring and are now considered pervasive in natural systems. Maternal effects have been widely studied in vertebrates, especially in the context of social environment, and are often mediated by steroid hormone (SH) deposition to eggs. In insects, although many species dramatically alter phenotype and life‐history traits in response to social density, the mechanisms of these alterations, and the role of hormone deposition by insect mothers into their eggs, remains unknown. In the experiments described here, we assess the effects of social environment on maternal hormone deposition to eggs in house crickets (*Acheta domesticus*). Specifically, we tested the hypotheses that variable deposition of ecdysteroid hormones (ESH) to eggs is affected by both maternal (a) social density and (b) social composition. We found that while maternal hormone deposition to eggs does not respond to social composition (sex ratio), it does reflect social density; females provision their eggs with higher ESH doses under low‐density conditions. This finding is consistent with the interpretation that variable ESH provisioning is an adaptive maternal response to social environment and congruent with similar patterns of variable maternal provisioning across the tree of life. Moreover, our results confirm that maternal hormone provisioning may mediate delayed density dependence by introducing a time lag in the response of offspring phenotype to population size.

## 1 INTRODUCTION

Social environment profoundly influences the fitness of animals, affecting their probability of survival to adulthood, longevity, and reproductive output (McCullough, 1999; Nieberding & Holveck, 2017; Siracusa et al., 2017). However, social environment can vary substantially over relatively short time scales as population sizes fluctuate, as age structure changes, or as seasonal processes alter the types of groups to which animals belong (Clutton‐Brock, Price, Albon, & Jewell, 1992; Lewellen & Vessey, 1998; Randall, Rogovin, Parker, & Eimes, 2005; Tinghitella, 2014). Therefore, social conditions experienced by parents at the time of reproduction can predict the social environments that offspring will face (Simpson & Miller, 2007). As a consequence, some adults express flexibility in their reproductive strategies in response to their social environment. For example, female cockroaches (*Nauphoeta cinearea*), increase the number of male, but not female, offspring that they produce when they encounter few males in their developmental and adult environments (Moore, Gowaty, Wallin, & Moore, 2001). As a consequence, they increase the fitness of their sons in a female‐biased environment. Similarly, female guinea pigs (*Cavea apera* f. *porcellus*) alter the sex ratio of their offspring to produce more daughters than sons when social conditions are unstable (Kemme et al., 2009). In this species, females reach reproductive maturity earlier than males, so a female‐biased sex ratio is interpreted to be adaptive in unstable conditions (Kemme et al., 2009).

Beyond simple changes in the sex ratio of their offspring, mothers may also alter the phenotypes of their offspring based on variation in social environment. Non‐Mendelian parental effects (hereafter, maternal effects) provide one mechanism by which the social environment experienced by parents can influence offspring phenotype, life history and fitness (reviewed in Mousseau and Fox (1998b) and Groothuis, Muller, von Engelhardt, Carere, and Eising (2005)). Social environment is a promising environmental variable to induce maternal effects, because while it can fluctuate within an animal's lifetime (Hamilton, 1966), a mother's social environment can also predict the social conditions her offspring will face (Dantzer et al., 2013). For such maternal effects to be adaptive, mothers must be able to predict at least some aspects of their future environments, and influence offspring phenotype accordingly (Carlisle, 1982; Dantzer et al., 2013; Schwabl, Mock, & Gieg, 1997). Despite clear challenges in predicting future environmental conditions, adaptive maternal effects are now considered pervasive in natural systems (Champagne, 2008; Chin et al., 2009; Mousseau & Fox, 1998b; Rossiter, 1996; Shea, Pen, & Uller, 2011).

Maternal effects on offspring life history have been widely studied in vertebrates (Beach, 1974; Burton, Hoogenboom, Armstrong, Groothuis, & Metcalfe, 2011; Craft et al., 2009; Maestripieri, 1993; Stein & Bell, 2014), particularly in avian systems, in which they are often mediated by steroid hormone (SH) deposition to eggs, most notably in the form of androgens and glucocorticoids (Love, Chin, Wynne‐Edwards, & Williams, 2005; McNabb & Wilson, 1997; Rutkowska & Badyaev, 2008; Tschirren, Richner, & Schwabl, 2004). Notably, the amount of SH in eggs can reflect maternal social environment: both the composition and size of social groups affect adult endocrine status and reproductive strategy. For example, female great tits (*Parus major*) allocate higher concentrations of androgens to eggs when breeding at high social densities (Remeš, 2011), which increases the growth rate of their sons, but not of their daughters after hatching (Tschirren, 2015). Similarly, female house sparrows (*Passer domesticus*) vary the concentration of androgens that they deposit to their eggs according to their social environment (Mazuc, Bonneaud, Chastel, & Sorci, 2003). Their offspring that are exposed to elevated androgen concentrations are more competitive (Strasser & Schwabl, 2004). In Japanese quail (*Coturnix coturnix*), increased glucocorticoid titers (a SH stress indicator that responds to social density) in mothers are transferred to eggs, reducing the growth rate of juvenile offspring and increasing the sensitivity of adult offspring to environmental stressors (Hayward & Wingfield, 2004). Maternal steroid hormones can therefore have adaptive or maladaptive effects on offspring phenotype and fitness, depending on the concentrations and specific hormones deposited into eggs.

Beyond vertebrates, research on insect systems has greatly informed our understanding of the evolution of social behavior and adaptation to variable social environments (Lester, Grach, Pener, & Simpson, 2005; Tibbetts & Izzo, 2010; Wilson & Wilson, 2007). As with vertebrates, the social environment experienced by parents can influence the phenotype of offspring. For example, females of both the *Delphacidae* species *Prokelisia marginata* and *P. dolus* respond to increased social density by altering the phenotype of their offspring: at low densities, offspring in both species are flightless, but in crowded conditions, they produce fully winged offspring that can disperse (Denno & Roderick, 1992). However, research on hormone‐mediated effects of parental social experience on offspring reproductive strategy remains scarce, largely because of the logistical issues associated with measuring the very small quantities of hormones present in insects and their eggs.

Of notable exception are studies on the infamously polyphenic locust species, *Schistocerca gregaria*: these studies are both numerous and extend to epigenetic maternal effects (reviewed in Maeno & Tanaka, 2010). Female desert locusts respond to social crowding by producing gregarious offspring, a polyphenic phase morphologically and behaviorally distinct from the solitarious phase in desert locusts (Maeno, Piou, Ould Babah, & Nakamura, 2013). While variable hormone deposition to eggs is not responsible for the shift between phases (Simpson & Miller, 2007), substantially higher concentrations of ecdysteroid hormones (ESH, steroid hormones ubiquitous in invertebrate species) are found in the fresh eggs of crowded females, compared to ESH concentrations in fresh eggs produced by solitary females (Hägele, Wang, Sehnal, & Simpson, 2004). Further, the eggs produced by crowded females produce larger hatchlings than those laid by isolated females, although they take longer to mature (Uvarov, 1966). These findings are consistent with our previous work on house crickets (*Acheta domesticus*), which has shown that a higher mass of ESH available to fresh eggs (less than 24 h old) results in larger hatchlings, despite uniform egg weight (Crocker & Hunter, 2018).

*Acheta domesticus* is an excellent system for studying hormone‐mediated maternal effects. Unlike those of the polyphenic *S. gregaria*, the general ecology and life history of *A. domesticus* do not depend on a polyphenic phase. Therefore, house crickets are suitable for investigating smaller‐scale, nuanced effects of variable hormone provisioning on offspring phenotype. Female house crickets provision their eggs with highly variable amounts of ESH in sufficient quantities to measure (Dinan, 1997). Moreover, variable social conditions influence orthopteran development, affecting adult phenotype, endocrine status and reproductive decisions in many species within the order (Bertram, Fitzsimmons, McAuley, Rundle, & Gorelick, 2012; Crankshaw, 1979; Hägele et al., 2004; Tinghitella, 2014; Woodring, Meier, & Rose, 1988).

*Acheta domesticus* is amenable to laboratory rearing, which makes it an expedient organism with which to investigate transgenerational effects of variable maternal hormone deposition to eggs. For example, we have previously shown that variation in egg ESH provisioning translates into variation in offspring life history traits, whereby increased ESH provisioning results in larger, faster‐growing hatchlings in spite of uniform egg size (Crocker & Hunter, 2018). Although our previous study identified one cause (grand‐maternal diet quality) of variation in ESH provisioning, this variable did not account for all of the variation that we observed. In this study, therefore, we ask whether social environment is an additional and important determinant of ESH provisioning in eggs.

We assessed the effects of social environment on maternal hormone deposition in house cricket eggs. We hypothesized that variable deposition of ESH to eggs is an adaptive maternal response to environmental stressors, because in a previous study, we found that female *A domesticus* appeared to negotiate an ESH provisioning/egg number trade‐off differently, based on the quality of diet available to their maternal grandmothers (Crocker & Hunter, 2018). In this study, we tested the hypotheses that variable deposition of ESH to eggs is affected by both (a) maternal social density and (b) maternal social composition. Addressing our first hypothesis, we predicted that in high social density, female crickets would deposit more ESH to their eggs (resulting in larger, faster‐growing hatchlings). We made this prediction because there is evidence that in many taxa, parents produce more competitive offspring at higher densities (Benton et al., 2005; Meylan, Miles, & Clobert, 2012), and because development rate in a cannibalistic species may been linked to the level of competition faced during development (Garay, Varga, Gamez, & Cabello, 2016). Regarding our second hypothesis, we predicted that female house crickets reared in a female‐skewed environment would deposit more ESH to their eggs (thus, producing larger, faster‐growing hatchlings) than would females reared in male‐skewed social environments. We made this prediction based on the observation that the number of reproductive females in a population is a better predictor of the population size of the subsequent generation than is the number of adult animals in that population.

We measured differences in ESH provisioning by female *A. domesticus* raised under varying social densities and compositions in a laboratory experiment. We found that while the ESH provisioned by mothers to their eggs does not reflect social composition (sex ratio), it does reflect social density. Specifically, female crickets reared at low population density provided higher doses of ESH to their eggs than did those raised in high population density, regardless of sex ratio. Maternal hormone provisioning of ESH to eggs thus links maternal social environment to the phenotype of her offspring.

## 2 METHODS

### 2.1 Experimental methods

We reared crickets following the general methods described by Crocker and Hunter (2018) and Tinghitella (2014), briefly summarized here. We ordered 1500 juvenile crickets from reptilefood.com, and raised them to maturity in a communal bin. After approximately a third of the crickets had reached sexual maturity (estimated visually by the appearance of wings), we provided a shallow dish of damp coconut fiber into which female crickets oviposited. After 4 days, we removed the dish and incubated it at 30°C, checking daily for hatchlings. We separated 4000 hatchlings into 160 perforated 12‐oz to‐go salad containers (Genpak AD12: 5.38″ long by 4.5″ wide by 2″ high): 120 containers had 30 hatchlings each (high‐density population), 40 containers had 10 hatchlings each (low‐density population) (both population densities were modeled after those described by Tinghitella (2014)). All containers were provided with food (ground Harlan Teklad Rodent Diet 8604) and water (in wet gravel‐filled 1‐oz plastic cups) ad libitum (modeled after Whattam & Bertram, 2011), and all densities were maintained throughout the experiment.

As crickets grew, we increased the size of their rearing containers, but maintained them at their experimental densities (Table 1). Every week, we counted hatchlings in each salad container: containers that had fewer than the 30 or 10 crickets necessary were combined to result in fewer overall replicates, but consistent population density. At no time in the experiment were any crickets switched between high‐ and low‐density treatments. When hatchlings were 3 weeks old, we transferred crickets from their original replicate containers to larger (32‐oz) perforated to‐go salad containers (Genpak AD32, 7.25″ long by 6.38″ wide by 2.63″ high), maintaining their experimental densities. Up until 6 weeks after hatching, food was provided by scattering an excess amount across the floor of the container daily: this was performed to ensure that all hatchlings would be able to locate and access food ad libitum, without needing to compete for it.

**Table 1:** Summary of experimental conditions. Group size refers to the number of crickets in each bin during an individual's lifetime. Columns labeled “Males per bin” and “Females per bin” refer to the group composition formed at 6 weeks after hatching. The replicates column shows the number of experimental replicates (bins) that were used for each social condition. The low‐density treatment had three focal females per bin while all other treatments had six. The column labeled “Total” indicates the number of samples collected for each social condition

Social condition	Group size	Males per bin	Females per bin	Replicates (N bins)	Focal females per bin	Total (replicates * focal females)
High density	30	15	15	6	6	36
Low density	10	5	5	12	3	36
High female	30	10	20	6	6	36
High male	30	20	10	6	6	36

Six weeks after hatching, but before maturation, crickets can be sexed (Crocker, personal observation): we then transferred crickets into their final experimental containers. For these containers, we used screen‐lidded 14‐L bins (after Tinghitella, 2014) in which we maintained cricket densities, but also created three different sex ratios for the high‐density crickets: equal, female‐skewed, and male‐skewed (Table 1); low‐density crickets were established at 50:50 sex ratios only. We created a total of 24 experimental bins, with the replicate number of bins per treatment provided in Table 1. These bins were provided with food ad libitum in 100 mm petri dishes, which were large enough for all crickets in the bin to eat simultaneously (thus minimizing the effect of any competition for food), and water in gravel‐filled 8 oz deli cups (after Whattam & Bertram, 2011). We stored all bins in a 6 × 4 array of soundproofed, subdivided shelves insulated with 2″ thick egg‐crate acoustic foam and SoundBreak XP Drywall.

After transferring crickets to the screen‐lidded bins, we checked all bins daily for crickets that had reached maturity. Six females from each high‐density bin, and three females from each low‐density bin, were chosen to serve as focal females in the experiment. To control for any confounding effects of a cricket's latency to mature relative to the population she interacted with, we chose focal females in all bins by the ordinal number in which they matured (e.g., in low‐density bins, the first, third, and fifth females to mature). For each of these focal females, we then calculated her age at maturity as the number of days after hatching at which she reached her imaginal molt (assessed visually by the presence of wings and fully formed ovipositor).

To distinguish focal females, we marked each cricket's pronotum with a dot of paint when they reached maturity: focal females were marked with a unique color; all other crickets were marked with white. Every evening, we isolated focal females individually in small screen cages within their respective bins. Overnight, focal females were provided with individual oviposition dishes of damp sand, and water. Nonfocal females were provided with a communal oviposition dish of damp sand, and water: food was removed from all bins overnight to control for any effect on focal females. Each morning after oviposition dishes were collected, focal females were released into the bin to eat and interact with conspecifics in their replicate bin. Female *A domesticus* lay multiple clutches (in the lab, 1–15) of eggs during their lives, and clutches range in size between 1 and 400 eggs (Crocker, personal observation).

We measured the size at maturity of each focal female by photographing her over a millimeter‐demarked ruler. Then, in Adobe PhotoShop 7.0, we measured each cricket's pronotum width (in pixels) as an indicator of structural size (Kelly, Tawes, & Worthington, 2014). To convert our pixel measurement to millimeters and standardize our measurements between crickets, we then measured the width of the photographed millimeter in pixels, and used this to convert the cricket measurement.

For each focal female, we recorded the length of time (days) between reaching maturity and laying their first batch of eggs (hereafter, lay latency). All eggs were collected as described by Crocker and Hunter (2018) and stored at −20°C (Warren et al., 2006). We used a subset (*N* = 20) of each focal female's eggs to quantify the mass of active ESH present per egg using an Enzyme Immunoassay protocol described previously (Crocker & Hunter, 2018). In brief, we partitioned ESH from eggs into methanol (MeOH), which we dried by vacuum. We then reconstituted the extract in 0.5M Tris‐HCl buffer, and purified it with 60% MeOH in Milli‐Q water elution across a C‐18 Solid Phase Extraction column. We dried the 60% MeOH solution under vacuum and dissolved the purified extract in EIA Buffer (Cayman Chemical Company, Ann Arbor, MI, USA), which we then used to assay ESH mass per sample via the Cayman Chemical Company's 20‐hydroxyecdysone enzyme immunoassay materials (see Crocker & Hunter, 2018 for details and a validation of this assay and extraction technique). For each female, we used eggs from the first clutch laid; in most cases (54 of 100 focal individuals, evenly dispersed across treatments (ANOVA, *F*_3,96_ = 1.50, *p* = 0.219)), we supplemented the sample using eggs from subsequent clutches. Previous experiments have shown that *A. domesticus* do not vary in their ESH provisioning of eggs over their lifetime (Crocker & Hunter, 2018), and so combining clutches for analysis will not alter the measured masses of ESH per egg.

### 2.2 Statistical methods

We used separate mixed linear models (SAS 9.0) to assess the impact of (a) social density and (b) social composition on maternal provisioning of ESH to eggs. When assessing effects of social density on the mass of ESH measured in eggs, we compared only those crickets reared under equal sex ratios (Table 1). When assessing effects of sex ratio on ESH, we compared only those crickets reared at high density (Table 1). ESH measurements were log‐transformed prior to analysis to meet conditions of homogeneity of variance and were the dependent variable in the models.

In analyses presented here, we have used the mass of ESH per egg as our dependent variable, reasoning the dose per offspring unit (i.e., dose per embryo) is the most physiologically relevant measure. However, we conducted the same analyses using mass of ESH per gram of egg weight (i.e., concentration) and we present these results in supplementary material (Supporting information Table S1): the analyses of ESH concentration yielded similar results to those of dose per egg. Social density (high/low) or sex ratio (equal, female‐skewed, male‐skewed) were fixed effects in the separate models. To assess whether a female's body size, age at maturity, or lay latency was associated with the amount of ESH that she provided to her eggs, we included these variables in our models. The unique identifier of the bin in which each female was raised was included as a random effect.

## 3 RESULTS

**Figure 1:**
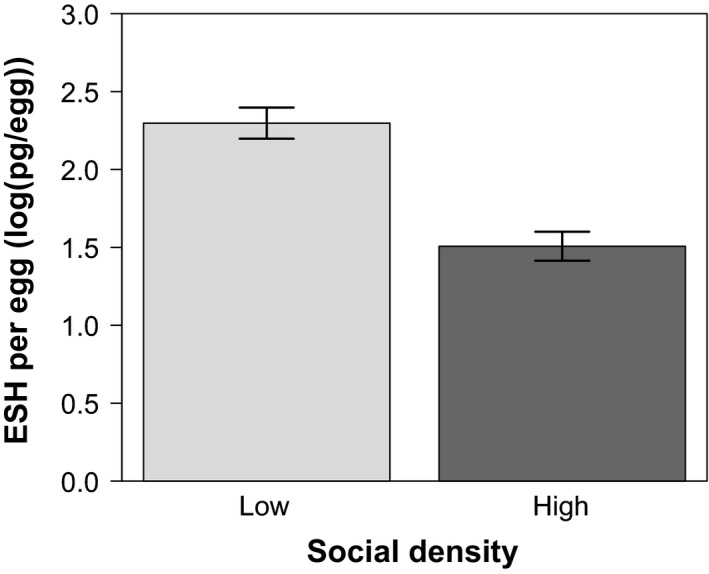
Bar plot depicting the mean masses of ESH provided per egg by *Acheta domesticus* females reared in high and low density environments (bars show standard error). Females reared in high density environments deposited less ESH in their eggs than did females reared in low‐density environments

**Figure 2:**
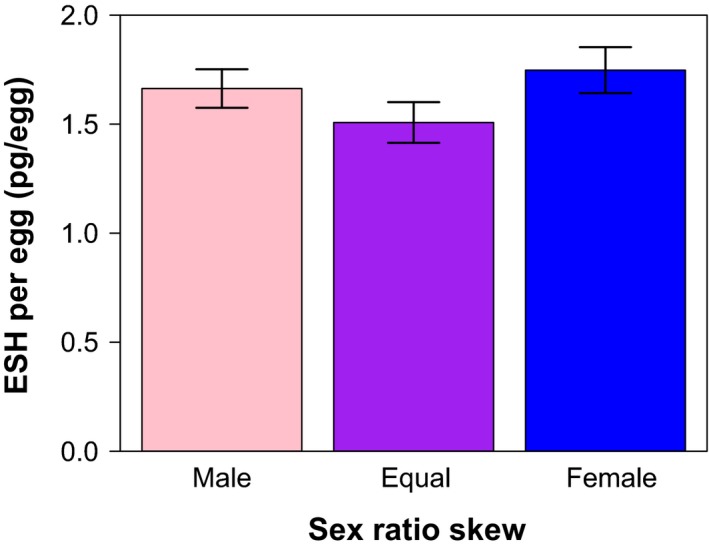
Bar plot depicting the mean masses of ESH that female *Acheta domesticus* deposited per egg under different social compositions (sex ratios) (error bars show standard error). Neither Male‐skewed (Male) nor Female‐skewed (Female) sex ratios induced females to deposit ESH in their eggs in concentrations that differed from females in equal sex ratio (Equal) environments

We provide averages of cricket traits (focal female size, ESH/egg, age at maturity, and lay latency) among treatment groups in Table 2, and the results of statistical models of variation in ESH/egg in Table 3. Female crickets provided higher doses of ESH to their eggs when raised at low density than they did when raised in high density groups (*F*_1,22_ = 12.53, *p* = 0.0018) (Figure 1, Tables 2, 3). In contrast, the sex ratio under which female crickets were reared from 6 weeks to adulthood had no measured impact on the ESH doses that they allocated to eggs (*F*_2,38_ = 0.05, *p* = 0.947) (Figure 2, Tables 2, 3). Under different sex ratios (though not between social densities), the provisioning of ESH to eggs by female crickets correlated positively with a female's age at maturity (*F*_1,38_ = 6.51, *p* = 0.0149, Supporting information Figure S3). However, this effect of age at maturity on ESH provisioning was absent when we analyzed ESH concentration rather than does per embryo (Supporting information Table S1).

**Table 2:** Effects of density and sex ratio (Composition) on female pronotum size, time to maturity, time between reaching maturity and initiating oviposition, and ESH provisioning per egg. Data are mean values and standard errors. We have included the results of post hoc statistical comparisons among the groups in each column; statistical similarity (α = 0.05) is denoted by the same letter superscript before each mean, while statistical difference (α = 0.05) is indicated by differing superscript letters. Note that the sample sizes here (in the column labeled “N”) differs from that in Table 1, because we selected a subset of focal females from the groups described by Table 1 to use in our molecular analyses below

Comparison	Treatment	*N*	Mean size (mm)	Avg ESH/egg (log(pg))	Age at maturity (days)	Lay latency (days)
Density	High	24	^a^4.83 ± 0.13	^a^0.655 ± 0.04	^a^70.59 ± 1.53	^a^18.68 ± 1.43
Density	Low	27	^a^5.01 ± 0.08	^b^0.998 ± 0.04	^a^68.04 ± 1.33	^a^18.93 ± 1.72
Composition	Equal	24	^a^4.83 ± 0.13	^a^0.655 ± 0.04	^a^70.59 ± 1.53	^a^18.68 ± 1.43
Composition	High female	26	^a^5.1 ± 0.08	^a^0.759 ± 0.04	^a^70.95 ± 1.82	^a^20.85 ± 1.39
Composition	High male	23	^a^4.7 ± 0.09	^a^0.722 ± 0.04	^a^69.10 ± 1.97	^a^19.4 ± 0.96

**Table 3:** Statistical models to assess potential drivers of variation in ESH provisioning of eggs by crickets. Each linear mixed model was initially run including all interaction terms, and nonsignificant interaction terms between fixed effects were removed individually (according to lowest value of Mean Squares) between iterations of the model. Fixed effects are included in the model line for each; the unique identifier of the bin each cricket was raised in was included in all models as a random effect

Model	ESH/egg ~ Density + Female Size + Lay Latency + Age at Maturity		
	Density	*F*_1,22_ = 12.53	*p* = 0.0018
	Female size	*F*_1,22_ = 0.00	*p* = 0.969
	Lay latency	*F*_1,22_ = 0.90	*p* = 0.353
	Age at maturity	*F*_1,22_ = 1.13	*p* = 0.299

Model	ESH/egg ~ Sex Ratio + Female size + Lay latency + Age at maturity		
	Sex ratio	*F*_2,38_ = 0.05	*p* = 0.947
	Female size	*F*_1,38_ = 0.38	*p* = 0.541
	Lay latency	*F*_1,38_ = 1.22	*p* = 0.277
	Age at maturity	*F*_1,38_ = 6.51	*p* = 0.015

## 4 DISCUSSION

Our results demonstrate that female house crickets reared at high density allocate lower doses of ESH per egg than do crickets reared at low density. Consistent with work on the related desert locust (*Schistocerca gregaria*) (Hägele et al., 2004), we predicted that we would observe a difference in provisioning strategy between crickets raised in high and low density treatments. Previous experiments in this same system have shown consistently that higher doses of ESH per egg result in faster juvenile growth rates (Crocker & Hunter, 2018).

Given the effect of ESH on growth, we can infer that crickets reared at low density will produce faster‐growing hatchlings, whereas crickets reared at high density will produce slower‐growing hatchlings, regardless of the diet quality available (Crocker & Hunter, 2018). In this experiment, we expected that crickets reared at higher social densities would prime their offspring to encounter competition by depositing higher doses of ESH into their eggs, thus producing larger offspring and speeding their development (Crocker & Hunter, 2018).

Our predictions were based on the large body of maternal investment work substantiating MacArthur and Wilson's *r*‐ and *K*‐selection framework (MacArthur & Wilson, 1967). Specifically, it has been broadly demonstrated that high parental density is associated with maternal effects that favor rapid offspring growth rates, presumably to accelerate consumption of declining resources (Burton et al., 2011; Dantzer et al., 2013; Denno & Roderick, 1992; Hayward & Wingfield, 2004; Kemme et al., 2009; Lester et al., 2005). Although a majority of these studies have been conducted with vertebrates. Benton et al. have demonstrated similar effects in populations of soil mites (Benton et al. 2005), which negotiate a trade‐off between egg size and the extent of protein a female deposits to each egg. In the studies of which we are aware, results have been interpreted as evidence of the fitness benefits of producing fewer, high‐quality offspring in low‐food (or high‐stress) conditions, and more, lower‐quality offspring in high‐food (or low‐stress) conditions (Dantzer et al., 2013; Meylan et al., 2012).

Strikingly, our results indicate that while a female cricket's ESH provisioning strategy responds to her social environment, it does so in the opposite direction to that we predicted (Figure 1, Table 3). The observed pattern of provisioning may be adaptive in the case of a population that oscillates around an ecologically optimal density (Witting, 2000). Female crickets under high density may program their offspring to grow more slowly in order to increase their likelihood of survival in resource‐limited conditions. For example, in stressful conditions, a reduced metabolic budget (which is associated with slower growth rates) can increase an invertebrate's likelihood of survival (Nespolo, Lardies, & Bozinovic, 2003; Wallace, 1973). This framework can also explain the higher provisioned level of ESH to eggs laid by females that experienced the low‐density social condition. At low population densities, fast‐growing individuals are more likely to gain higher fitness than (and even cannibalize (Crocker, personal observation)) smaller conspecifics.

Density experienced by mothers has been found to alter the number of offspring they produce, in addition to the phenotype and growth of those offspring (Benton et al., 2005). For example, female gypsy moths (*Lymantria dispar*) detect increased social densities via altered phytochemicals in the leaves on which they feed, and respond by programming their offspring to become heavier at pupation (Rossiter, 1991). Following this same pattern, females of both the *Delphacidae* species *Prokelisia marginata* and *P. dolus* respond to increased social density by altering the phenotype of their offspring: at low densities, offspring in both species are flightless, but in crowded conditions, they produce fully winged offspring that can disperse (Denno & Roderick, 1992). Most importantly, in the desert locust (a close relative of house crickets) crowded females produce fewer, larger eggs than solitary females, and optimize their offspring to survive starvation conditions (Maeno et al., 2013).

In this study, we observed no effect of social composition on the provisioning of ESH by female crickets to their eggs. In these comparisons (i.e., under no variation in density) females that were older when they matured provided more ESH to their eggs (Supporting information Figure S3). This finding was not replicated in our analyses using ESH concentration (Supporting information Table S1), which can likely be explained by the finding that females who were older whey they matured produced heavier eggs (Supporting information Figure S4). However, we suggest that this phenomenon should be measured in more detail in a study powered to detect the effects of female age on ESH provisioning.

We acknowledge that our methods unavoidably use a shorter treatment for the social composition treatment groups than for the social density treatment groups. In light of other studies citing changes to the behavior of female Gryllids in response to alterations to the sex ratio on much shorter time scales, (Lehmann, 2007; Souroukis & Murray, 1994), we argue that this does not diminish the robustness of results reported here. We also note that we are unable to account for any selective pressures on survival in different social environments that may be acting in our experiment, because we did not measure the mortality rate of hatchlings.

Our data are consistent with the interpretation that variable ESH provisioning is an adaptive maternal response to social environment, and maternal provisioning of ESH may be a mechanism that generates delayed density dependence in populations. These results are congruent with similar patterns of variable maternal provisioning in response to environmental stressors that have been observed in other taxa (Reviewed in Mousseau and Fox (1998a,b), and Uvarov (1966); see Dantzer et al. (2013), Schwabl et al. (1997), and Rossiter (1991) for selected examples). However, more work is needed in this area. For example, if our conclusions are correct, we predict that an intermediate population density of crickets would result in an intermediate level of ESH provisioning to eggs.

Variable maternal hormone provisioning is a promising context in which to investigate adaptation to variable environments (Dantzer et al., 2013; Jablonka et al., 1995; Meylan et al., 2012; Mousseau & Fox, 1998a; Schwabl et al., 1997; Witting, 2000), particularly given current rates of environmental change. Finally, density‐mediated variation in egg hormone provisioning provides a potential mechanism underlying the time‐lagged effects of parental environment on offspring life history that can destabilize population dynamics and generate outbreaks (Ginzburg & Taneyhill, 1994; Rossiter, 1991).

## COMPETING INTERESTS

We have no competing interests.

## AUTHOR CONTRIBUTIONS

KCC and MDH conceived the study design and general methods, KCC wrote funding proposal and carried out experiments with input from MDH, KCC wrote manuscript in collaboration with MDH.

## DATA ACCESSIBILITY

Data will be made available with manuscript, and archived in the Dryad repository, https://doi.org/10.5061/dryad.jf75dq3.
