# Supplementary material for: Social density, but not sex ratio, drives ecdysteroid hormone provisioning to eggs by female house crickets (Acheta domesticus)
Source: Ecol Evol. 2018 Oct 2;8(20):10257–65. doi: 10.1002/ece3.4502 (PMC6206184; doi:10.1002/ece3.4502)
Supplement: Supplementary file 1 [file ECE3-8-10257-s001.docx]

Supplementary Materials

Social density, but not sex ratio, drives ecdysteroid hormone provisioning to eggs by female house crickets (*Acheta domesticus*)

Katherine C. Crocker & Mark D. Hunter

Social environment profoundly influences the fitness of animals, affecting their probability of survival to adulthood, longevity, and reproductive output. The social conditions experienced by parents at the time of reproduction can predict the social environments that offspring will face. Despite clear challenges in predicting future environmental conditions, adaptive maternal effects provide a mechanism of passing environmental information from parent to offspring, and are now considered pervasive in natural systems. Maternal effects have been widely studied in vertebrates, especially in the context of social environment, and are often mediated by steroid hormone (SH) deposition to eggs.

In insects, although many species dramatically alter phenotype and life history traits in response to social density, the mechanisms of these alterations, and the role of hormone deposition by insect mothers into their eggs, remains unknown. In the experiments described here, we assess the effects of social environment on maternal hormone deposition to eggs in house crickets (*Acheta domesticus*). Specifically, we tested the hypotheses that variable deposition of ecdysteroid hormones (ESH) to eggs is affected by both maternal (1) social density and (2) social composition.

We found that while maternal hormone deposition to eggs does not respond to social composition (sex ratio), it does reflect social density; females provision their eggs with higher ESH doses under low density conditions. This finding is consistent with the interpretation that variable ESH provisioning is an adaptive maternal response to social environment, and congruent with similar patterns of variable maternal provisioning across the tree of life.

Here we have included additional information and analyses. In Table 1S, we provide the results of models which used the mass ratio of ESH per egg as the dependent variable. These results match our general findings presented in the main text.

Figure S1 shows that the mass of cricket eggs (measured in batches of twenty eggs) does not vary with social density. As a consequence, the variation in ESH per egg that we observed under variable social density (main text) does not derive changes in the mass of individual eggs that we used for analysis.

Figure S2 illustrates that a female’s structural size was not a driver of the weight of the eggs she produced. Taken together with Figure 1S, this supports our suggestion that potential competition for food (or its potential outcome—smaller body size at maturity) is not driving the differences in provisioning of ESH that we note as our main result. Rather, female crickets may respond to their perception of social density as they provision their eggs.

Table S2 is an expansion of Table 2 in the main text: it shows F statistics and p values for the summary statistics presented in the main text.

Figure S3 is a scatterplot illustrating the effect of a female’s age at maturity on the amount of ESH she provided to her eggs under different sex ratios. Importantly, though this result was statistically significant in the analysis we did which used dose of ESH per egg as the response variable, when we controlled for egg weight (by using ESH mass per mass egg; that is, concentration) this result disappeared (Table 1S).

Figure S4 is a scatterplot illustrating the effect of a female’s age at maturity on the weight of the 20-egg group that we analyzed for ESH content. This result is likely the reason why we did not find any result for a female’s age at maturity in models in Table 1S. However, we suggest caution in interpreting this result and encourage the completion of a study powered to explicitly test female age at maturity as a potential cause of variation in egg mass.

| **Table S1:** Statistical models to assess potential drivers of variation in ESH provisioning of eggs by crickets using the mass of ESH per milligram of eggs analyzed. Each model was initially run including all interaction terms, and non-significant interaction terms were removed individually (according to lowest value of Mean Squares) between iterations of the model. No interaction terms were of statistical significance. |
| --- |
| \| *Model* \| *g ESH / g egg ~ Density + Female Size + Lay Latency + Age at Maturity* \| \| \| \| --- \| --- \| --- \| --- \| \|  \| *Density* \| F_1,22_ = 11.41 \| p = 0.003 \| \|  \| *Female Size* \| F_1,22_ = 1.57 \| p = 0.223 \| \|  \| *Lay Latency* \| F_1,22_ = 0.10 \| p = 0.752 \| \|  \| *Age at Maturity* \| F_1,22_ = 0.29 \| p = 0.598 \| \| *Model* \| *g ESH/ g egg ~ Sex Ratio + Female Size + Lay Latency + Age at Maturity* \| \| \| \|  \| *Sex Ratio* \| F_2,37_ = 0.34 \| p = 0.712 \| \|  \| *Female Size* \| F_1,37_ = 0.32 \| p = 0.577 \| \|  \| *Lay Latency* \| F_1,37_ = 0.13 \| p = 0.725 \| \|  \| *Age at Maturity* \| F_1,37_ = 1.05 \| p = 0.312 \| |
|  |
| **Figure S1:** Bar plot depicting the mean (and SE) mass of 20 egg aliquots used in our hormone analyses for focal females raised under low or high social density treatments in our experiment. (F_1,49_ = 1.145, p = 0.29) |
|  |
| **Figure S2:** Scatterplot depicting the weights of the 20 egg aliquots used in our hormone analyses as a function of the structural size of the female that laid them (N = 88, F_1,86_ = 1.848, p = 0.178). |
| **Table S2:** Table of expanded model statistics for Table 2 in the main text. Here we provide the F statistics and p values that underlie the statistical comparisons denoted by letter superscripts in Table 2 of the main text. |
| \| Model \| F statistic \| p value \| \| --- \| --- \| --- \| \| *Female size ~ Density* \| F_1,44_ =1.35 \| 0.252 \| \| *Age at maturity ~ Density* \| F_1,46_ = 1.6 \| 0.212 \| \| *Lay latency ~ Density* \| F_1,49_ = 0.54 \| 0.466 \| \|  \|  \|  \| \| *Female size ~ Sex ratio* \| F_2,59_ = 3.00 \| 0.057 \| \| *Age at maturity ~ Sex ratio* \| F_2,59_ = 0.30 \| 0.743 \| \| *Lay latency ~ Sex ratio* \| F_2,70_ = .094 \| 0.91 \| |
|  |
| **Figure S3:** Color-coded scatterplot showing the relationship between a female’s age at maturity (x-axis) and the mass of ESH she provides to her eggs (y-axis). Only females from high-density bins are shown: magenta squares show females from male-skewed sex ratio bins, purple squares show females from equal sex ratio bins, and blue squares show females from female-skewed sex ratio bins. Females that were older at maturity provided more ESH per egg than females that matured at a younger age (R^2^ = 0.068, N = 62, F_1,60_ = 5.47, p = 0.023). |
| **** |
| **Figure S4:** Scatterplot showing the relationship between a female cricket’s age at maturity (x-axis) and the mass of 20 eggs that we analyzed. (Only females from high-density bins are shown). Females that delayed maturity produced heavier eggs (R^2^ = 0.078, N = 61, F_1,59_=6.05, p = 0.017). |
